# Thoracic Computed Tomography Findings in Malignant Mesothelioma

**DOI:** 10.5812/iranjradiol.8764

**Published:** 2012-11-20

**Authors:** Omer Tamer Dogan, Ismail Salk, Fikret Tas, Kursat Epozturk, Cesur Gumus, Ibrahim Akkurt, Sefa Levent Ozsahin

**Affiliations:** 1 Department of Chest Diseases, Faculty of Medicine, Cumhuriyet University, Sivas, Turkey; 2 Department of Radiology, Faculty of Medicine, Cumhuriyet University, Sivas, Turkey

**Keywords:** Asbestos, Tomography, X-Ray Computed, Mesothelioma, Cystic, Thorax

## Abstract

**Background:**

Malignant pleural mesothelioma (MPM) is an uncommon neoplasm. MPM occurs more frequently in patients born or living in certain villages of Turkey.

**Objectives:**

We aimed to review radiological findings of MPM.

**Patients and Methods:**

We reviewed the CT findings in 219 biopsy-proven MPM patients admitted to our clinic between 1993 and 2008.

**Results:**

The most common CT findings included pleural thickening (n=197, 90%) classified as diffuse (n=138, 63%), nodular (n=49, 22%) and mass-type (n=16, 7%). Pleural effusion was found in 173 patients (79%), involvement of the interlobar fissures in 159 (73%), mediastinal pleural involvement in 170 (78%), volume contraction in 142 (65%), mediastinal shift in 102 (47%) and mediastinal lymphadenopathy in 54 (25%).

**Conclusion:**

MPM may present with diverse radiological features. Pleural thickening and pleural effusion were the most frequent radiological findings. Thoracic CT scans might be assessed more cautiously in patients with environmental exposure to asbestos.

## 1. Introduction

Malignant pleural mesothelioma (MPM) is an infrequent neoplasm (1). However, MPM is the most common primary malignancy of the pleura and its incidence is estimated as 2000-3000 cases per year in the United States of America (2, 3). MPM occurs frequently in patients from certain villages in the central and southeastern regions of Turkey compared to other parts of the country. Nowadays, thoracic computed tomography (CT) scan with contrast is a much more sensitive examination for diagnosing, staging and follow-up of patients with MPM (4-9). CT plays an important role in the assessment and diagnosis of these tumors and is superior to conventional chest radiography (10).

## 2. Objectives

In this study, we evaluated CT findings in 219 cases of MPM caused by environmental exposure to asbestos fibers.

## 3. Patients and Methods

We retrospectively reviewed the CT scans of patients with biopsy-proven MPM evaluated at our clinic between 1993 and 2008. All patients had a history of environmental asbestos exposure. All patients had a chest radiograph taken maximum two-weeks before computed tomography. Thoracic CT was performed in all subjects, using a Picker PQS (Cleveland, OH, USA) spiral CT scanner. Evaluation was carried out in chest images obtained in 10 mm slices from the apices of the lung to costophrenic angles. The sections were taken in the supine position at the end of the inspiration. Intravenous iodinated contrast medium was given to the patients to determine mediastinal pathologies. Patients were prospectively evaluated with CT scan and the sections were evaluated by a radiologist blind to pathological results, to give binary decisions. No rating scale was utilized. The diagnoses of malignant mesothelioma were confirmed pathologically in all cases. In most patients, the diagnosis of mesothelioma was made by closed pleural biopsy; in others, the diagnosis was reached through transthoracic biopsy, thoracoscopy, thoracotomy, cytologic examination, extrathoracic biopsy and pericardiectomy. The pleural thickening was classified as diffuse, mass type and nodular and the localization of pleural effusion as unilateral and bilateral. In cases of different types of pleural thickening in the same individual, each type was noted separately. Diffuse pleural thickening was demarcated as a pleural thickness of 10 mm or less, pleural nodules as focal pleural thickness of 10-30 mm and pleural masses as lesions of 30 mm or more in diameter. Involvement of interlobar fissures and mediastinal pleura were noted. The presence of calcified pleural plaques and hyaline pleural plaques on the contralateral pleura was also assessed. Hyaline pleural plaques were defined as a focal increase in soft tissue density along the pleura, which is well demarcated and clearly separated from the lung. Dislocation of the mediastinal structures was defined as “mediastinal shift”. The volume loss of hemithorax was defined as volume contraction. If mediastinal lymph nodes were greater than 10 mm, they were considered as pathological. Both hemithoraces were evaluated for pulmonary parenchymal abnormalities such as tumoral invasion or fibrosis and presence of calcified pleural plaques. All these findings were recorded for both males and females (11-15).

## 4. Results

The study included 219 malignant pleural mesothelioma patients (129 men and 90 women). The mean age of the patients was 58.3 ± 12.6 years, with a range of 18-85 years. Nearly half of these patients were coming from Yildizeli, a town in Sivas and the villages around this town where the risk of environmental exposure to asbestos is high. The rest of the patients were mainly residents of Sivas and surrounding cities. Eighty-two patients (37.4%) were current smokers; the others included former, passive, and never smokers. The average of cigarette smoking history among all smokers was 27.93 ± 21.59 pack-years. The results are presented in Tables 1 and 2.

**Table 1:** CT Findings in 219 Patients with MPM

	Male	Female	Total, No. (%)
**Pleural Thickening**			
**Diffuse**	81	57	138 (63.0)
**Irregular**	30	15	45 (20.5)
**Mass type**	48	36	84 (38.3)
**Smooth**	28	21	49 (22.4)
**Nodular**	10	6	16 (7.3)
**Pleural Effusion**			
**Unilateral**	94	74	168 (76.7)
**Bilateral**	3	2	5 (2.3)
**Interlobar Fissural Involvement**	95	64	159 (72.6)
**Mediastinal Pleural Involvement**	96	74	170 (77.6)
**Pleural Plaque**			
**Hyaline**	21	11	32 (14.6)
**Calcified**	3	3	6 (2.7)
**Mediastinal shift**	59	43	102 (46.6)
**Volume Contraction**	88	54	142 (64.8)
**Mediastinal Lymphadenopathy**	35	19	54 (24.7)
**Atelectasis**	28	17	45 (20.5)
**Chest Wall Involvement**	20	15	35 (16.0)
**Pneumothorax**	2	1	3 (1.4)
**Parenchymal Abnormalities**	18	10	28(12.8)

**Table 2:** Chest X-Ray Findings in 219 Patients with MPM

	No. (%)
**Pleural Thickening**	105 (47.9)
**Pleural Effusion**	146 (66.7)
**Volume Contraction**	94 (42.9)
**Mediastinal Shift**	70 (32.0)
**Pleural Calcifications**	24 (11.0)
**Thickening of Interlobar Fissure**	26 (11.9)
**Pneumothorax**	3 (1.4)

## 5. Discussion

CT plays an important role in the diagnosis and assessment of patients with mesothelioma. Pleural thickening and pleural effusion are the most encountered features. Although some findings are quite characteristic, none is pathognomonic for the disease. Nevertheless, CT remains to be the dominant modality for assessing patients with mesothelioma, including evaluation of treatment response. CT findings can delineate the optimal site for biopsy, while providing a tremendous amount of anatomic information about the stage of the disease (16-20).
